# Transcriptomic Response under Heat Stress in Chickens Revealed the Regulation of Genes and Alteration of Metabolism to Maintain Homeostasis

**DOI:** 10.3390/ani11082241

**Published:** 2021-07-30

**Authors:** Hana Kim, Hyeran Kim, Pilnam Seong, Devender Arora, Donghyun Shin, Woncheoul Park, Jong-Eun Park

**Affiliations:** 1 Division of Animal Genomics and Bioinformatics, National Institute of Animal Science, Wanju 55365, Korea; hanakim0307@gmail.com (H.K.); devarora@korea.kr (D.A.); wcpark1982@korea.kr (W.P.); 2 Animal Nutrition and Physiology Team, National Institute of Animal Science, Wanju 55365, Korea; ococ1004@korea.kr (H.K.); spn2002@korea.kr (P.S.); 3 Department of Agricultural Convergence Technology, Jeonbuk National University, Jeonju 54896, Korea; sdh1214@gmail.com

**Keywords:** heat stress, chicken, MAPK signaling pathway, calcium signaling pathway, transcriptome analysis

## Abstract

**Simple Summary:**

With the increased global temperature, the threat from climate change has already affected the livestock industry. Exposure to heat stress is a major factor responsible for impacts on the overall livestock production, which ultimately results in economic losses. With no exception, poultry is among the most vulnerable livestock to environmental stress. Hence, a comprehensive study is required to understand the molecular mechanisms and to improve the breeding program to overcome economic losses. Therefore, we investigated growth related phenotypes and performed transcriptome analysis to understand the heat stress response in chickens. Animal experiments were designed with two groups, which were kept at 21 and 33 °C for 2 weeks as the control and treatment groups. The transcriptome analysis used blood samples from each chicken. In this study, we identified a total of 245 differentially expressed genes (DEGs) with important roles in various biological processes, such as cell protection, energy conversion in the mitochondria, and protein quality control. The results indicate that the heat stress environment regulates genes and alter the metabolism to adjust for the heat environment in chickens. These findings could be useful to help understand the heat stress response in poultry.

**Abstract:**

Chicken is important livestock that serves as a vital food source which remain largely affected by heat stress. Therefore, we performed the transcriptome analysis to help understand the mechanisms of heat stress response in chickens. In the animal experiments, we grouped them into a normal and severe at 21 and 33 °C, with identified physiologic parameters for 2-weeks. Subsequently, RNA-seq analysis was performed to identify DEGs with a false discovery rate < 0.05 and a fold change ≥ 1.5. In the physiological parameters, we observed average daily gain was declined, rectal temperature and respiration rate was increased in severe group. Among total 245 DEGs, 230 and 15 genes were upregulated and downregulated, respectively. In upregulated DEGs, HSPs, *MYLK2,* and *BDKRB1* genes were identified as key genes in heat stress. The KEGG pathway analysis showed involvement in the ATP metabolic process, MAPK signaling pathway and calcium signaling pathway with related protein processing and synthesis. In conclusion, with induced heat stress, such changes in physiologic parameters alter the neuroendocrine system, and we observed that the heat stress environment regulates such Heat shock protein genes to protect the cells and proteins from an altered metabolism. These findings provide a more comprehensive understanding of the heat stress response in poultry.

## 1. Introduction

Animal husbandry practice involves breeding, farming, and care of farm animals. Higher production in poultry farming mainly depends on nutrient intake, climatic condition, and surrounding humidity [1]. In recent years, we have witnessed a change in climatic conditions, and an increased average global earth temperature, which ultimately leads to global warming. Chicken breeds are divided into layers and broilers, where a layer chicken is an important livestock animal that lays eggs as an important protein source in the food industry, and a broiler chicken is a major breed that is bred for meat, and contributes to about 34.3% of the global meat production [2]. In the last decade, husbandry practices for chickens have been drastically affected by heat stress, as high temperatures have led to physiological changes, and exposure of these high temperatures to broiler chickens has decreased feed intake [3,4]. The calcium level in chickens is known to have an important role in eggshell mineralization, and high temperature affects the calcium conditions, such as the eggshell shape, character, thickness, and weight. Moreover, it also impacts the subfertility, which leads to a decline egg production [4,5,6]. Such a response incurs a decline in products, decreased growth rate, body weight, and meat quality [2]. Altogether, it results in economic losses, panting in poultry, high body temperature, increased plasma corticosterone, oxidative stress, and inhibition of the immune response, which are known physiological changes by heat stress [3,4,5,6,7]. Additionally, it was reported that heat stress on poultry resulted in economic losses of $128 to $165 million in a year [2,8,9]. Hence, effort should be made to cope with the detrimental effects of heat stress on poultry.

In poultry farms, the optimum temperature for growing chicken breeds varies from 18 to 22 °C. Induced heat stress, due to increased terrestrial temperature, is a visible threat directly affecting the poultry physiology and immune system [10]. These physiological changes lead to alterations of the neuroendocrine system, decreased feed intake, increased respiration rate, and loss of water when exposed to a heat environment [2,6,11,12,13]. The altered neuroendocrine system has an important role in maintaining homeostasis and helps to regulate hypothalamic-pituitary-adrenal (HPA) and sympathetic-adrenal medullar (SAM) axis [10]. Common physiologic parameters, such as feed intake, is regulated by the HPA axis in the neuroendocrine system; the SAM axis affects the immune response caused by a heat stress environment [12]. Hence, a heat stress environment is known to cause trouble, especially in laying chickens and broilers; thus, heat stress environments damage sensitive poultry [7]. However, there is limited evidence on the mechanisms underlying the decrease in poultry production due to this heat stress. In a previous study, heat stress on poultry was shown to alter production and cause physiological and economic losses, although, the reason was not completely determined for the decrease in production and its mechanisms; therefore, it requires further investigation to understand the role of heat stress in poultry production [10,14,15].

The heat shock protein (HSP) family and heat shock factors (HSF) are the typical genes that respond to heat stress, and their functions are reported in different studies [7]. Studies have suggested that upregulated HSP is involved in cell homeostasis, which involves responding to inflammation, immunity, and oxidant stress by heat stress. Among different HSPs, the HSPA (*HSP70*) gene is the most extensively studied member and widely used as a stress biomarker in the HSP family of genes [16,17,18]. The HSPA multigene family including *HPA1A*, *HPA1B*, *HSPA8,* and *HSPA5* have a critical role in reducing extracellular damage by altering gene expression and have an important role in immunity function and homeostasis [17,19,20,21]. HSP regulates the expression by HSF and is found in other animals, such as mammals and fish, and even poultry [20]. HSPA protects the cells against heat shock and oxidative stress-induced cell death through unclear mechanisms like in other animals and poultry. Additionally, HSP has an important role in pathway signaling, which protects the proteins from heat stress [22]. In previous studies, HSP was shown to alter gene expression to create a defense against heat stress, such as involving the reconstitution of gene expression. Pathway signaling and alterations in gene expression after exposure to stress is prudently regulated [22,23].

The HSP gene signaling pathways involved in HSP induction includes mitogen-activated protein kinase (MAPK) genes [24]. A strong association between HSPA gene expression and the MAPK signaling pathway has been reported in previous studies [25,26]. The mechanisms of the immune system responding to heat stress are still unclear as well as its role in cell protection and the involvement of HSP gene expression under certain conditions [27,28]. MAPK cascades have been classified into four types in mammalians; the MAPK components are extracellular signal-regulated kinase 1 and 2, c-Jun N-terminal kinase, p38, and *ERK5*. Among the MAPK cascades, extracellular signal-regulated kinase 1 and 2, c-Jun N-terminal kinase, and p38 have been reported to correlate with the HSP genes in previous studies. Moreover, MAPKs, which activate due to the stress response with HSPA, regulate intracellular substrates through phosphorylation to execute their functions [26]. With limited understanding, it has been reported that the upregulated HSAP genes are involved in the MAPK signaling pathways, although the role of the HSPA genes in the MAPK signaling pathways in heat stress is unclear in animals [29,30].

Therefore, a detailed analysis is required for further investigation to understand the detailed mechanisms underlying MAPK and the HSPA role in the heat stress mechanism. In this regard, this study was conducted to identify the responsible genes in heat stress and the genetic pathways through RNA sequencing analysis in the ROSS 308 chicken breed. This study will contribute to a better understanding of the transcriptomic mechanism of heat stress in chickens.

## 2. Materials and Methods

### 2.1. Animal Experiments

The animal experiments were performed following ethical guidelines, approval number 2020-429, in the National Institute of Animal Science (NIAS). In the pre-experimental period (1 to 13 days of age), female broilers (Ross 308 breed) were housed in batter cages and fed a standard commercial diet. At 14 days of age, a total 100 of broilers were divided into two environmentally controlled chambers. Taking into consideration their average body weight, the broilers were kept in individual metabolic cages (40 × 30 × 60 cm; length × depth × height) and acclimated to the chambers for 3 days under a thermos neutral condition (21 °C/60%), as described in previous studies [31,32]. After the adaptation period, the broilers were fed a standard commercial diet meeting all nutritional requirements and had ad libitum access to water and the feed for 2 weeks from when they were 17 to 31 days old. During the experiment period, the animals were reared in NIAS in chambers that controlled the temperature and humidity. After acclimation, the animals were divided randomly into a Normal and Severe group, and the temperature and humidity of the Normal group were kept following the previously suggested THI (Temperature–Humidity Index) method at 21.9 ± 1.1 °C and 68 ± 2.5% humidity (THI 69). The Severe group under heat stress were always kept at 32.9 ± 0.4 °C and 60.3 ± 2.0% humidity (THI 84) to the end of the experiments [33]. Moreover, 10 broilers per treatment were randomly selected for measuring the rectal temperature and respiration rate. The temperature was measured by inserting a rectal thermometer to a depth of 3 cm into the rectum. Respiration rate per minute was measured by the counting the number of breaths of the birds using a stop-watch. Subsequently, 12 random blood samples were drawn through a wing vein from 7 in the Normal and 5 in the severe group, and stored in a Tempus Blood RNA tube (Life Technologies, Carlsbad, CA, USA) for RNA isolation.

### 2.2. RNA Isolation and Sequencing

From each blood sample, RNA was isolated using the TRIzol:Chloroform method following the manufacturer’s instructions. The extracted RNA was checked for its integrity and purity and measured with the Agilent 2100 Bioanalyzer and the RNA Nano 6000 Assay Kit (Agilent Technologies, Santa Clara, CA, USA). Samples with RIN (RNA integrity number) values > 8 were used for library construction. The library was constructed with random cDNA fragments and acquired adapter-fragments of the cDNA using the TruSeq Stranded Total RNA LT Sample Prep Kit (Illumina, San Diego, CA, USA), following the manufacturer’s instructions. The constructed library was used to perform sequencing on the Illumina HiSeq 2500 platform and paired-end reads were generated. Finally, the generated BCL image files were converted to FASTQ raw reads using the bcl2fastq Illumina package which were used for the post-analysis.

### 2.3. Quality Control, Mapping, Counting and Batch Correction

First, a quality check was performed for the raw reads using FastQC v0.11.5 (released 8 March 2016, https://www.bioinformatics.babraham.ac.uk/projects/fastqc), and the trimming of N base and <Q20 reads was done using Trimmomatic v0.39 for quality control when generating the raw reads using the paired-end option [34]. Next, the filtered reads were used to make the index file, and mapping was done with the chicken (GRCg6a) reference-based genome using Hisat2 v2.2.1 [35]. The mapped reads were used to generate a count matrix using FeatureCounts of the Subread package v2.0.1 [36]. After counting the mapped reads, batch correction was performed, and any unwanted factors in the reads were removed using the RUVr function, which considers residuals from a first pass general linear model (GLM) regression, of the RUVSeq v1.24.0 package in R [37]. The corrected counts were converted to log_2_ + 1 values to check the correction. Then, using the calculated PC value, the after correction effect with the before correction was identified using the principal component analysis (PCA) plot. Finally, each corrected count file was used for the differential expression genes (DEGs) analysis.

### 2.4. Differentially Expressed Genes Analysis

We used the edgeR package v3.32.0 in R v4.0.3 to detect the DEGs between the two groups [38]. The normalization and the estimated dispersion values were acquired after performing the Cox–Reid profile-adjusted likelihood method in the correction stage. All genes were considered as DEGs having a false discovery rate (FDR) < 0.05 and fold change (FC) ≥ 1.5 genes [39]. The acquired DEGs were used for a heatmap to visualize the differential genes between the two groups. The DEGs were divided into upregulated and downregulated genes following the FC value. The PCA plot, bar plot, and heatmap used ggfortify v0.4.11 (released 3 October 2020, https://github.com/sinhrks/ggfortify), ggplot2 v3.3.3 (released 4 January 2021, https://cran.r-project.org/web/packages/ggplot2/ggplot2.pdf), and pheatmap package v1.0.12 (released 4 January 2019, https://cran.r-project.org/web/packages/pheatmap/pheatmap.pdf) in R for visualization.

### 2.5. Gene Ontology, KEGG Pathway, and Network Analysis

The Database for Annotation, Visualization and Integrated Discovery (DAVID) web tool was used for the enrichment analysis of the identified DEGs, and gene annotation was performed for the molecular functions, cellular components, biological processes, and pathways of the genes [40,41]. In DAVID, based on the Gene Ontology (GO) and Kyoto Encyclopedia of Genes and Genomes Pathway (KEGG) databases, Gallus species were selected by category and database, and GO databases were selected using the default settings [41,42]. Search&Color Pathway was performed in the KEGG Mapper of the KEGG, which showed different colors for each DEG based on the expression levels of the DEGs. The related genes with a top pathway were found with the ClueGO plugin in the GO and KEGG databases, which visualizes the biological term analysis and functionally grouped networks [42,43].

### 2.6. Statistical Analysis

All statistical analyses were performed with an adjusted *p* < 0.05. The physiological parameter of the animals was compared in the two groups by *t*-test, and DEGs were compared as previously described.

## 3. Results

### 3.1. Physiologic Parameters

**Figure 1:**
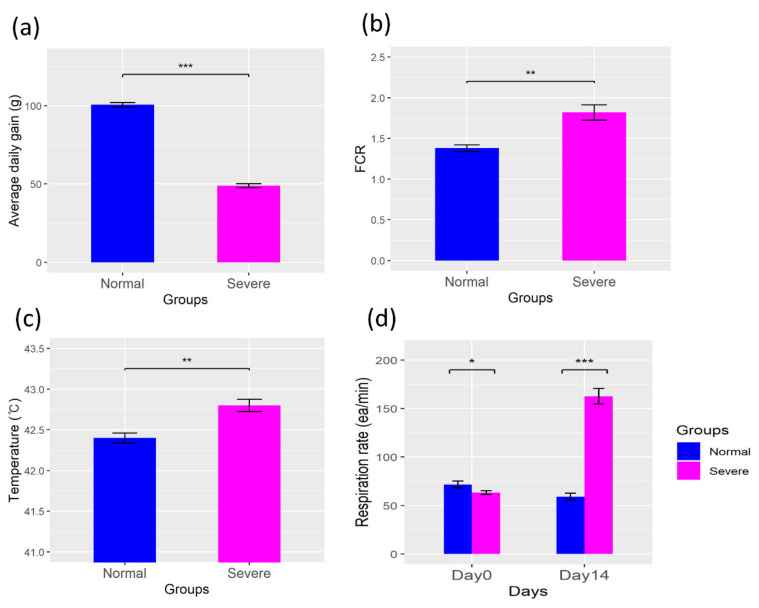
Comparison of the physiologic parameters between the two groups. (**a**) Average daily gain, (**b**) feed conversion ratio (FCR), (**c**) rectal temperature, (**d**) respiration rate. * *p* < 0.05, ** *p* < 0.01, *** *p* < 0.001, means ± S.E.

The chicken broilers were divided into the Normal and Severe groups for the normal and heat stress conditions, respectively, for a time period of 2 weeks, and daily monitoring was done for the following parameters: the average daily gain, feed conversion ratio (FCR), respiration rate, and rectal temperature. The results indicate a decline in the average daily gain in the Severe group when compared with the Normal group (*p* < 0.05). Here, the Severe group was observed to have a reduced average daily gain with an increased FCR, respiration rate, and rectal temperature shown in Figure 1. In particular, we found that the respiration rate of the Severe group was increased on day 14 (Figure 1d). Similarly, we saw a sharp decline in the average daily gain in the Severe group when compared with the Normal group (Figure 1a).

### 3.2. Construction of the Raw Reads, Mapping, and Batch Correction

**Figure 2:**
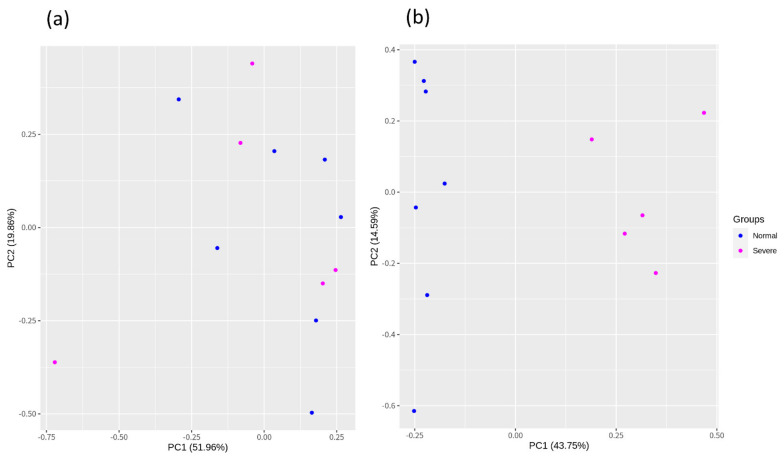
The batch correction using the RUVr function of the RUVSeq package; principal component was further divided into two groups: (**a**) before batch correction and (**b**) after batch correction using RUVr.

From a total of 12 samples, 1.7 to 2.2 G raw reads were generated. The Q20 and Q30 percentages were 92.73 to 98.43% and 87.05 to 95.42%, and the CG content percentage was 45.1 to 59.45% in each sample. The detailed information of the raw reads is provided in Supplementary Table S1. The mapping of the reads was performed using hisat2, and the mapped percentage was 60.07 to 93.27% reads with the chicken (GRCg6a) reference-based genome. Subsequently, we performed batch correction using the RUVSeq package to remove unwanted variations. Finally, the corrected data are shown in Figure 2.

### 3.3. Identification of DEGs

**Figure 3:**
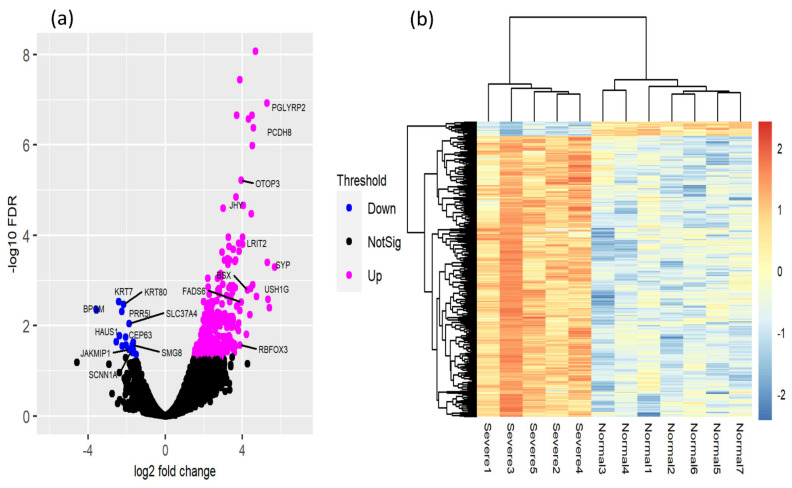
(**a**) Volcano plot showing the detected genes, DEGs, and the top 10 expressed genes involved in regulation between the two groups, (**b**) DEGs heatmap showing the detected DEGs and samples. The red and blue colors of the expression patterns indicate upregulated and downregulated, respectively.

A total of 371 DEGs was detected using edgeR with FDR < 0.05 and log_2_FC ≥ 1.5 as the upregulated and downregulated genes out of 24,356 genes. Among 371 RNAs, we identified 245 categorized into mRNAs, and among them, 230 were upregulated, and 15 were downregulated. The other 126 DEGs were 68 lncRNA, 54 novelgene, 3 pseudogenes, and 1 miRNA (provided in Supplementary Table S2). The DEGs and top 10 expressed genes are shown in Figure 3A. The heatmap for the expression patterns and the clustering for the DEGs and samples are shown in Figure 3B. Here, we report the top five upregulated and downregulated DEGs: upregulated, *SYP* (Synaptophysin), *PGLYRP2* (peptidoglycan recognition protein 2), *PCDH8* (protocadherin 8), *USH1G* (USH1 protein network component sans), and *LOC100859850*, and downregulated, *BPGM* (bisphosphoglycerate mutase), *KRT7* (keratin 7), *PRR5L* (Proline rich 5 like), *KRT80* (keratin 80), and *CEP63* (centrosomal protein 63). Additionally, the *MYLK2* (myosin light chain kinase 2), *FGFR1* (fibroblast growth factor receptor 1), and *DHDH* (dihydrodiol dehydrogenase) genes were expressed with an FC > 2.0, which include the *HSP* genes in the principal pathways. The results of the detected gene expressions with their FDR values are provided in Supplementary Table S2.

### 3.4. Function and Pathway Analysis of the DEGs

**Figure 4:**
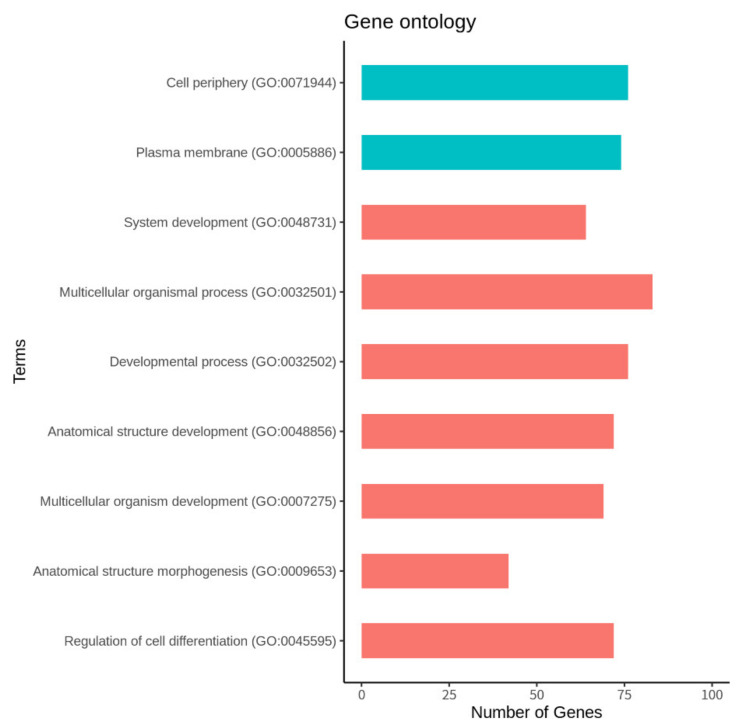
Enriched gene ontology. The green and coral colors of the functional annotation denote the cellular components and biological processes, respectively.

The identified 245 DEGs were used to perform GO analysis for functional annotation. The cutoff of the FDR < 0.05 was set to identify significant genes, and the DAVID server revealed that a total of seven biological processes and two cellular components were involved. The functional annotation found for various biological processes includes regulation of cell differentiation (GO:0045595), anatomical structure morphogenesis (GO:0009653), system development (GO:0048731), multicellular organism development (GO:0007275), anatomical structure development (GO:0048856), developmental process (GO:0032502), and multicellular organismal process (GO:0032501). Similarly, the functional annotation found for the cellular components was the plasma membrane (GO:0005886) and cell periphery (GO:0071944). The detailed annotation results are provided in Figure 4 and Supplementary Table S3.

**Figure 5:**
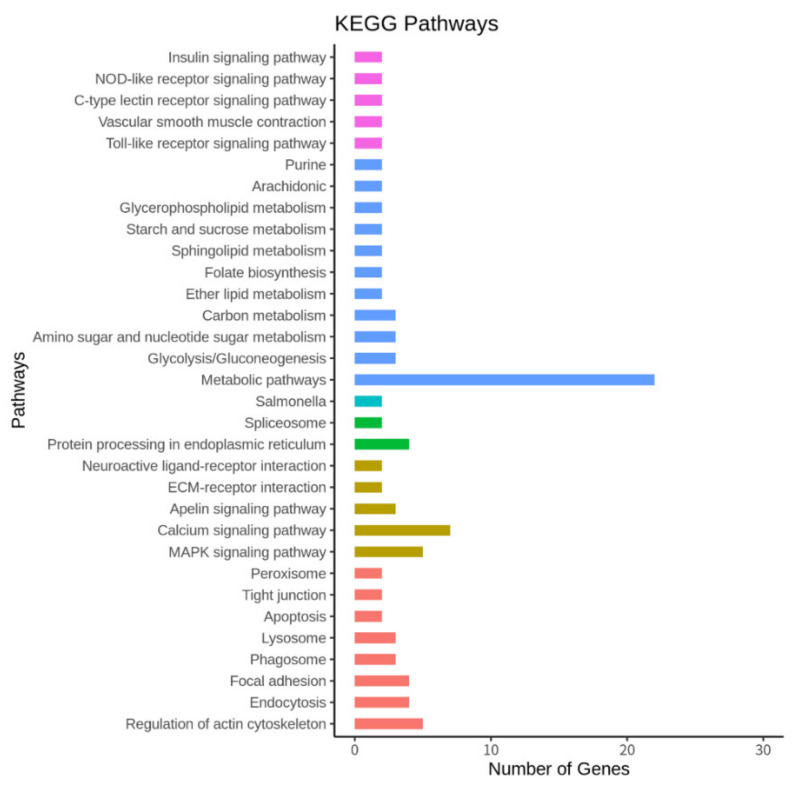
Enriched KEGG pathways of the DEGs (*p* < 0.05) denoted by different colors for the various pathway types and by the number of DEGs.

Later, we performed KEGG pathway analysis to elucidate the molecular interactions and biological functions of the DEGs. The identified KEGG pathways, shown in Figure 5, Supplementary Table S4, and Supplementary Figure S1, with the enriched top 10 pathways, were the metabolic pathways, MAPK signaling pathway, calcium signaling pathway, regulation of the actin cytoskeleton, endocytosis, focal adhesion, protein processing in the endoplasmic reticulum, glycolysis/gluconeogenesis, phagosome, amino sugar, and nucleotide sugar.

### 3.5. The Top 10 KEGG Pathways

**Figure 6:**
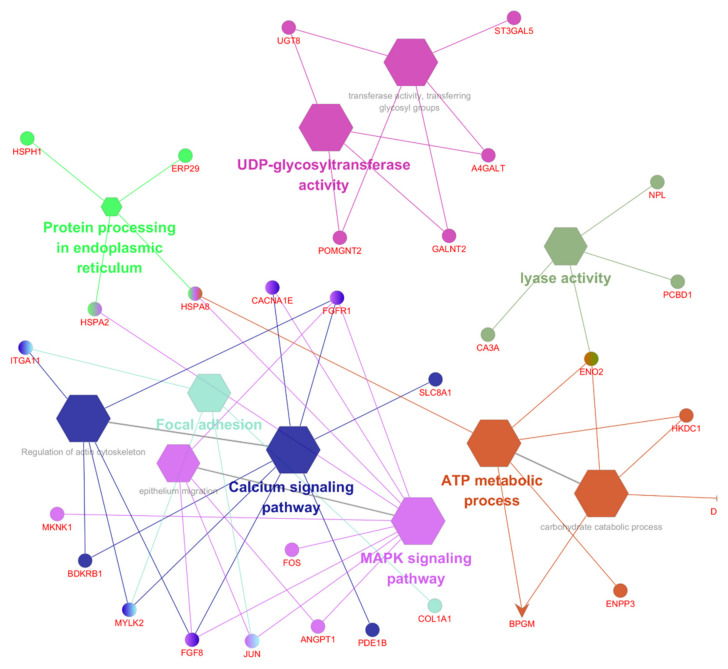
Gene network of the top 10 KEGG pathways with the elliptical shape representing the upregulated genes and the V shape representing the downregulated genes, respectively.

To select the top 10 enriched pathways representing the gene network, we set the parameters, such as the FDR value for the correction using the hypergeometric test and Benjamini–Hochberg (FDR < 0.05 and FC ≥ 1.5). The upregulated and downregulated genes are represented as elliptical shapes, and the pathways are represented in different colors (Figure 5). The represented enriched pathways belonged to the following: UDP-glycosyltransferase activity, carbohydrate catabolic process, transferase activity/transferring glycosyl groups, lyase activity, ATP metabolic process, epithelium migration, MAPK signaling pathway, calcium signaling pathway, protein processing in the endoplasmic reticulum, focal adhesion, and regulation of actin cytoskeleton pathways to upregulate genes and BPGM gene to downregulate genes (Figure 6).

## 4. Discussion

This study investigated the effect of heat stress on chicken broilers. The chickens were kept in the controlled humidity 68 ± 2.5% and 60.3 ± 2.0% for the Normal and Severe groups to affect the statistical analysis during post-processing of the data, kept the temperature as the one source of variance, and monitored the two main factors, which are the physiological and transcriptomic changes during heat stress [2,14,15].

### 4.1. Changes in the Physiological Parameters in a Heat Stress Environment

During the analysis, we confirmed that environmental heat stress leads to a change in the physiological parameters as observed in our animal experiments using 17-day old ROSS 308 female chickens in the THI 1 and four conditions for 2 weeks. Overall, the results showed significant changes between the two groups in the average daily gain, and the FCR was found to gradually decrease, whereas the respiration rate and rectal temperature were increased in the Severe group at the higher temperature. Similarly, the increased environmental temperature caused an increased body temperature. To prevent heat emission, the chicken homeostasis decreased the feed intake and body weight in the Severe group compared to the Normal group. Especially, an increased respiration rate alters the physiological response. As typical behavior in a heat stress environment, a severe respiration rate results in the loss of heat, and homeostasis keeps the body temperature from rising [10,12]. Additionally, exposure to high temperatures alters the neuroendocrine system in poultry. It activates epinephrine, nor-epinephrine, and plasma corticosterone, which increase muscle tone and nerve sensitivity and decrease protein synthesis. Thus, a severe respiration rate due to a heat stress environment degrades the muscles and affects the feed intake and body weight of chicken broilers as in previous studies [44,45,46]. Furthermore, the SAM and HPA axis activation in the neuroendocrine system, the release of glucose in the blood, and the decreased glycogen in liver and muscle induce protein synthesis and degradation to maintain homeostasis [46]. A previous study reported there was no relationship between alterations of the neuroendocrine system by heat stress and nutrient uptake although our results suggest that the average daily gain seems to be affected by a decrease in protein synthesis from exposure to a heat environment in the Severe group [10,44]. Therefore, exposure to a heat stress environment leads to a decrease in production by changes in the physiological parameters and neuroendocrine system in poultry. In conclusion, our results suggest that heat stress has a critical role in altering the physiologic parameters in ROSS 308 female chickens, which leads to a decrease in the FCR and average daily gain. These changes in the physiologic parameters are factors that decrease the production yield and cause economic losses.

### 4.2. Transcriptomic Changes in a Heat Stress Environment

In this study, we performed transcriptome analysis to confirm the regulation pattern of DEGs in a heat stress environment. We prepared a cDNA library and performed RNA sequencing for each blood sample; during the analysis, we detected 245 mRNAs transcripts with 230 upregulated and 15 downregulated mRNAs. GO and KEGG pathway analysis that elucidates the molecular interactions and biological functions of the DEGs showed nine GO terms and 32 enriched KEGG pathways. The results also show that the enriched genes were mainly involved in the adenosine tri-phosphate (ATP) metabolic process, MAPK signaling pathway, calcium signaling pathway, protein processing in the endoplasmic reticulum and focal adhesion. Especially, the MAPK signaling pathway, calcium signaling pathway, and focal adhesion are shown to share several genes, and the HSP genes were shown to be shared between the ATP metabolic process and protein processing in the endoplasmic reticulum pathway.

The identified genes contribute to the metabolic process known to have a crucial role in generating ATP for cellular energy conversion and protein synthesis in the mitochondria. Heat stress disturbs this metabolic process and affects the skeletal muscles and influences protein synthesis by altering the mitochondrial morphology [47,48,49]. Similarly, the endoplasmic reticulum has an important role in protein processing because it is associated with protein folding, assembly and translation functions in normal conditions [47,50,51,52]. In previous studies, both the ATP metabolic process and protein processing in the endoplasmic reticulum pathway were found linked with the regulation of several HSP genes [53]. In a stress environment, expressing HSP genes maintains the homeostasis of cells, by controlling protein denaturation using protein chaperons, and ATP hydrolysis to prevent protein aggregation. Therefore, it appears that the enriched ATP metabolic process pathway prevents denaturation of proteins as misfolded proteins through the activation of ATP hydrolysis, which mainly leads to the production of chaperons through the up-regulation of the HSP genes [54]. Protein synthesis from the HSP genes in the mitochondria decreases due to heat stress inducing variations in intracytoplasmic constructions, which causes a decrease in ATP production. Hence, there appears to be a correlation between the ATP metabolic process and the protein processing in the endoplasmic reticulum pathway through ATP hydrolysis through the regulation of the HSP genes [47,54]. Growth-related genes have been reported as downregulated when exposed to various stresses, whereas the upregulated HSP genes have been reported to protect cells and to have a heat-resisting property through an underlying mechanism, which needs to be fully understood under heat stress [16,23,55]. Additionally, HSP genes have been reported to control protein quality by regulating ATP hydrolysis energy and the enriched ATP metabolic process and protein processing in the endoplasmic reticulum pathways, which are involved in the upregulation of the HSP genes to protect cells and proteins during exposure to heat stress [17,54,56]. Therefore, we consider heat stress to affect the enriched ATP metabolic process and protein processing in the endoplasmic reticulum pathways by up-regulating the HSP genes.

Among the identified genes, the MAPK cascade is known as the central signaling pathway mainly responsible for regulating and responding to stress and for maintaining cell harmony by controlling essential cellular processes [28,57,58,59]. Whereas genes involved in the calcium signaling pathway are known to regulate immune cells and cell growth, development, proliferation, and gene expression [60,61,62]. Similarly, focal adhesion kinase is a mediator for cytoplasmic tyrosine kinase protein that connects the cytoskeleton to the extracellular matrix and has been reported to regulate cell migration; moreover, focal adhesion has been suggested to induce MAPK activity as reported in previous studies [63,64,65]. Altogether, these three pathways contribute to cell processing, which are found to be enriched during heat stress conditions [29,66,67]. Such a result appears to support a correlation between the MAPK signaling pathway, calcium signaling pathway, and focal adhesion involved in the cytoskeletal composition and cellular growth, and up-regulating the HSP genes induces the activation of the MAPK signaling pathway [61,62,65]. Especially, the Bradykinin receptor B1 (*BDKRB1*) gene, which generally is minimally expressed during normal conditions, has an important role in the calcium signaling pathway, in the response to inflammation, and in tissue damage [68,69,70]. Furthermore, the *MYLK2*, *FGFR1,* and *DHDH* genes were upregulated as DEGs. These genes were enriched in the calcium signaling pathway, MAPK signaling pathway, and ATP metabolic process, and these genes are known to be involved in the development of tissues, skeletal muscles, and the nervous system, and are overexpressed in response to inflammation [71,72,73,74,75]. Thus, we consider these enriched pathways to maintain cell homeostasis. In our results, we observed the *BDKRB1* gene differentially expressed due to heat stress which led to tissue damage. Moreover, HSP genes were upregulated to protect the cells, which leads to reconstruction of gene expression in response to the changing environment. The existing literature supports our study results because these genes are mainly involved in changing the gene expression patterns involved in metabolic alterations, mRNA synthesis, cell-type differentiation, cellular transport, and the cytoskeleton; those similar pathway genes were upregulated in our study [16,17,23]. Thus, cells resist a heat stress environment by metabolic alterations, cellular processes, and cell–cell connections through the cytoskeleton accomplished by changing the gene expression patterns. However, the role of the HSP genes in the MAPK signaling pathway is still unclear in animals. Additionally, there are no studies showing that the *BDKRB1* gene expression is upregulated in inflammation and tissue damage; the *BDKRB1* gene in the absence of the Bradykinin receptor B2 gene was found to be constitutively expressed; therefore, further investigation is required to decode the role of these identified genes [29,68,70]. In conclusion, the heat stress response leads to enriching the ATP metabolic process pathway, protein processing in the endoplasmic reticulum, MAPK signaling pathway, calcium signaling pathway, and focal adhesion. The upregulated HSP genes are involved in protecting the cells, including cellular energy conversion in the mitochondria and protein quality control, in addition to changing the gene expression pattern to the required one necessary for countering heat stress environments.

## 5. Conclusions

Two factors were mainly responsible for heat stress-triggered economic losses due to less production. First, alterations in the physiological parameters led to decreased body weight, average daily gain, FCR, respiration rate, and rectal temperature in ROSS 308 female chickens. The second factor responsible was the changed expression pattern of genes in response to heat stress, and in our observations, the genes involved in the ATP metabolic process, MAPK signaling pathway, calcium signaling pathway, protein processing in the endoplasmic reticulum, and focal adhesion pathway were enriched, and the heat stress response was the upregulation of the HSPs, *MYLK2* and *BDKRB1* genes. However, considering just the temperature condition in animal experiments requires further investigation to understand the causal effects of heat emission on the internal body. The results of this study suggest the MAPK signaling pathway is enriched, and HSP genes are upregulated in respond to heat stress, and those alter the metabolic pathway, which maintains homeostasis via cellular energy conversion and protein control. Therefore, this study contributes to a better understanding of the physiological parameters, gene expression, and metabolic pathways affected during the heat stress response in poultry.

## Data Availability

The data presented in this study are available on request from the corresponding author.

## Supplementary Materials

The following are available online at https://www.mdpi.com/article/10.3390/ani11082241/s1, Figure S1: The detailed results of the KEGG pathway analysis, Table S1: Information of the raw reads, Table S2: Information of the DEGs, Table S3: The detailed annotation results of the GO analysis, Table S4: The detailed results of the KEGG pathway analysis.
