# Supplementary material for: Transcriptomic Response under Heat Stress in Chickens Revealed the Regulation of Genes and Alteration of Metabolism to Maintain Homeostasis
Source: Animals (Basel). 2021 Jul 30;11(8):2241. doi: 10.3390/ani11082241 (PMC8388523; doi:10.3390/ani11082241)
Supplement: Supplementary file 1 [file animals-11-02241-s001.zip › Supplementary Figure 1.pptx]

## Slide 1
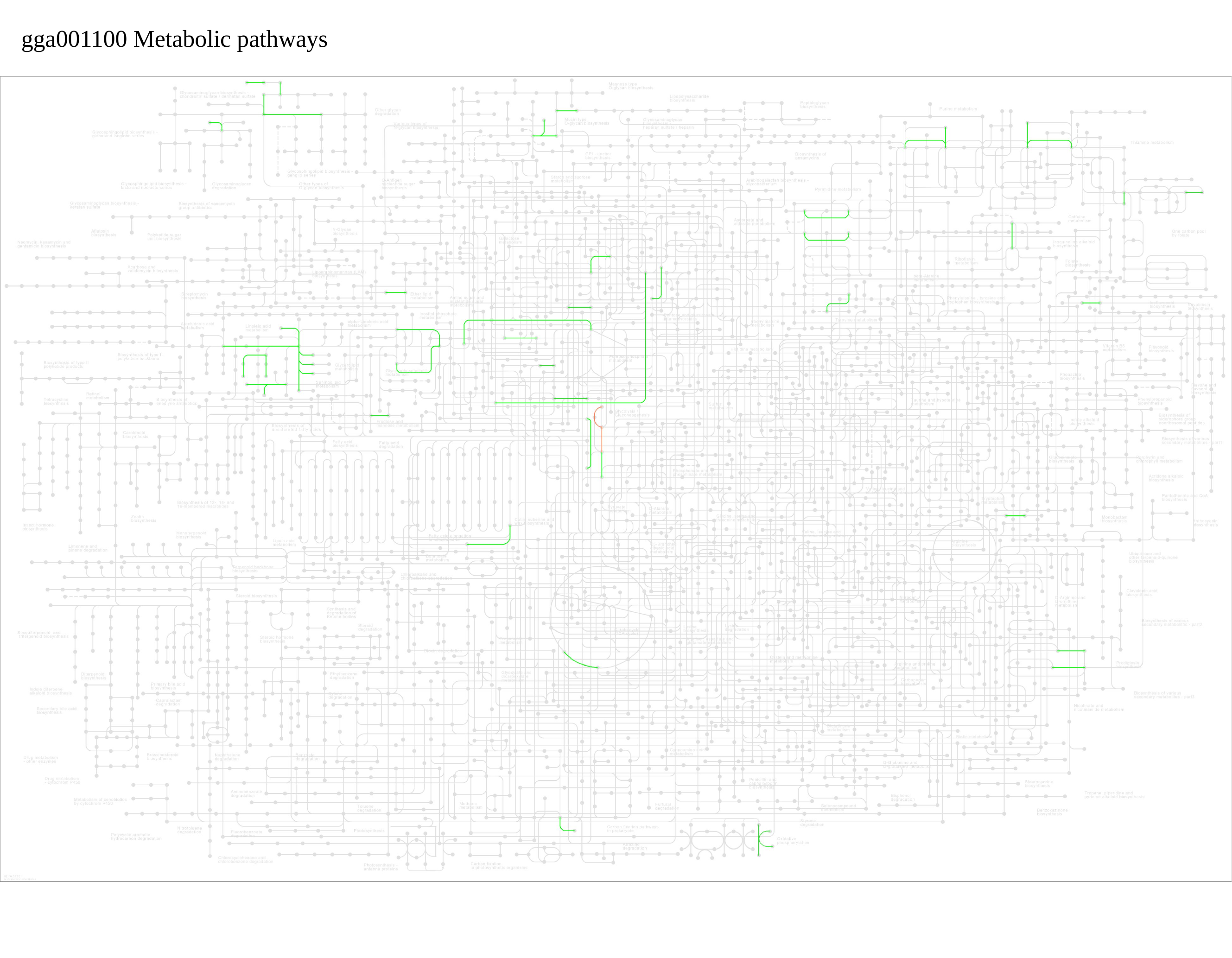

gga001100 Metabolic pathways

## Slide 2
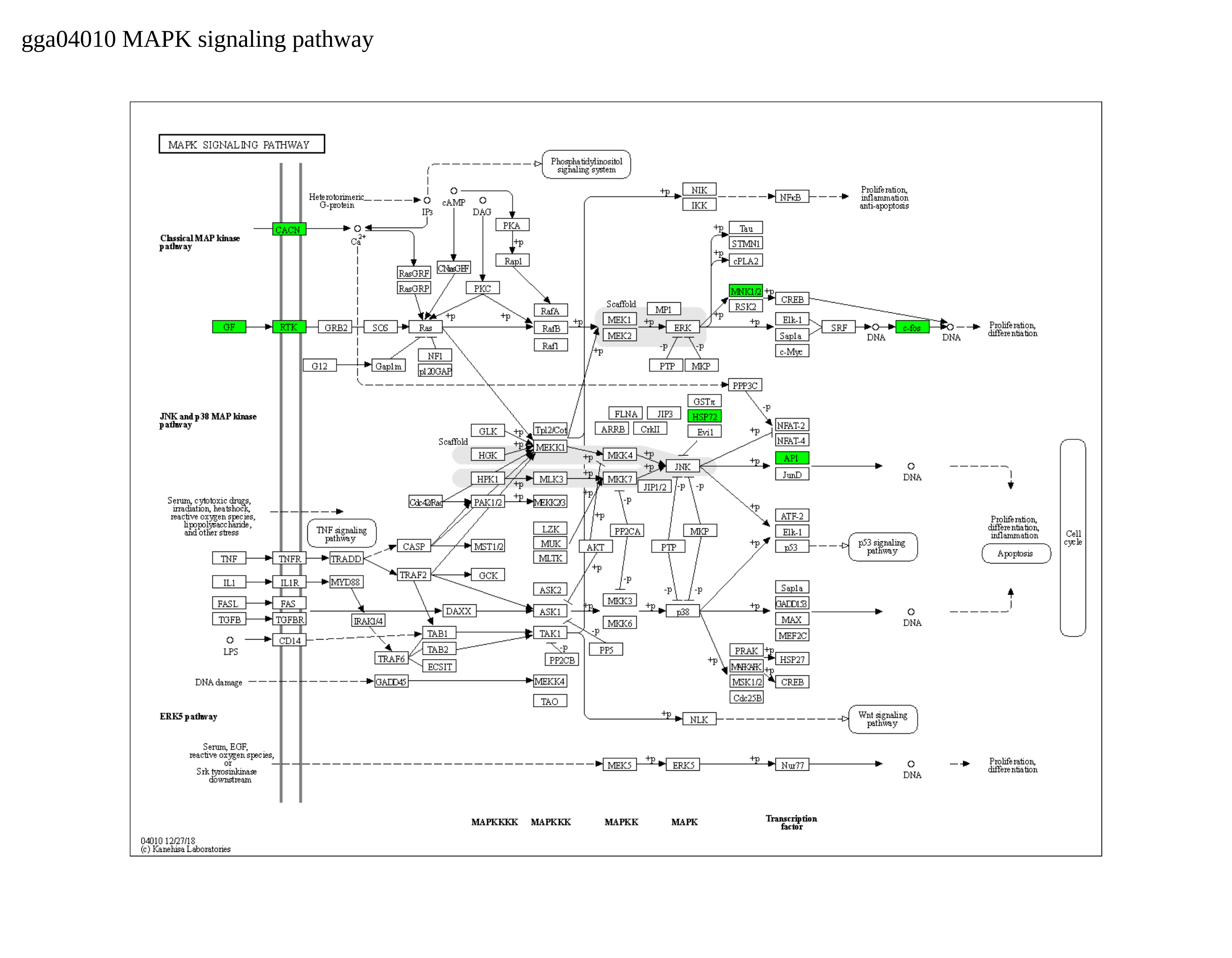

gga04010 MAPK signaling pathway

## Slide 3
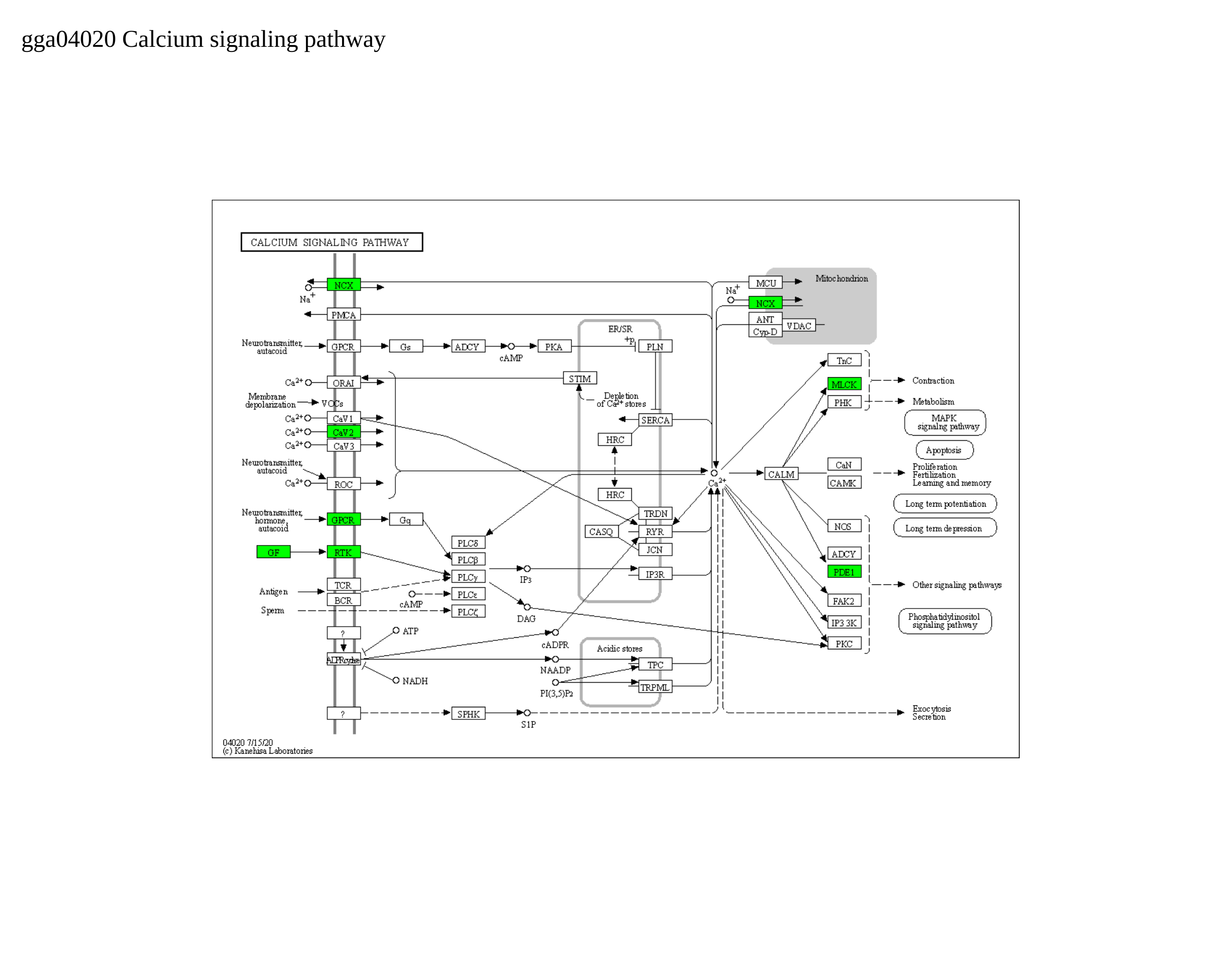

gga04020 Calcium signaling pathway

## Slide 4
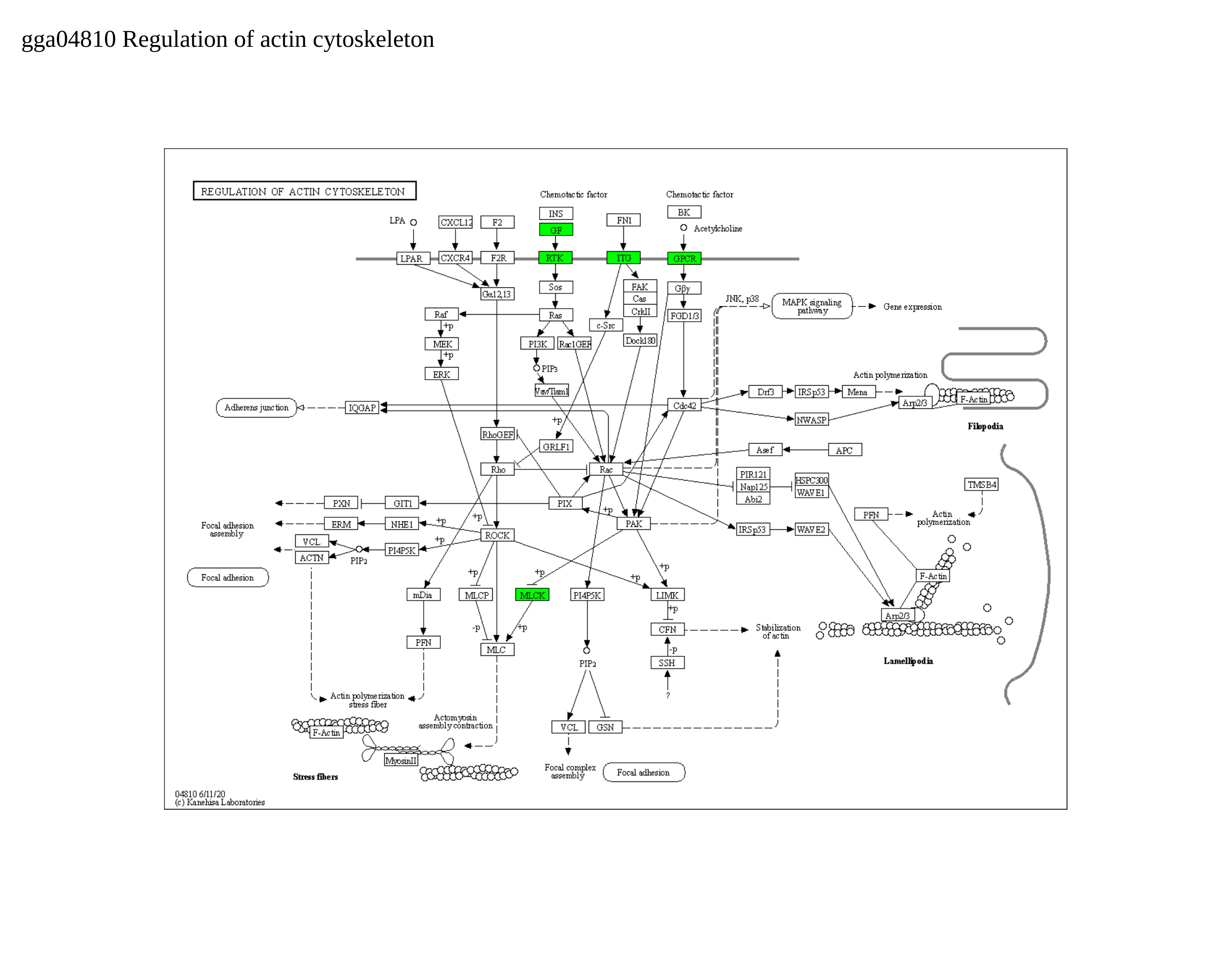

gga04810 Regulation of actin cytoskeleton

## Slide 5
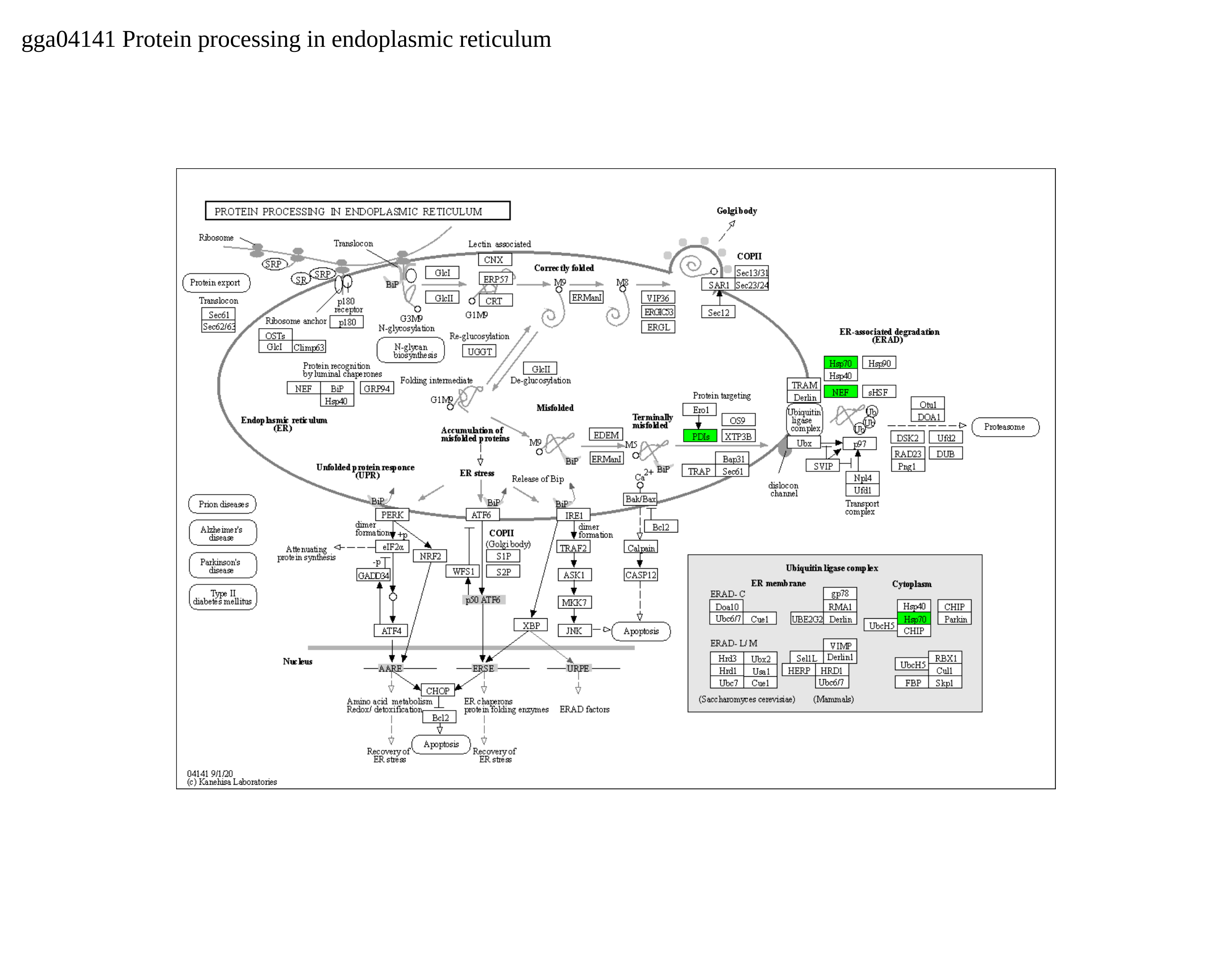

gga04141 Protein processing in endoplasmic reticulum

## Slide 6
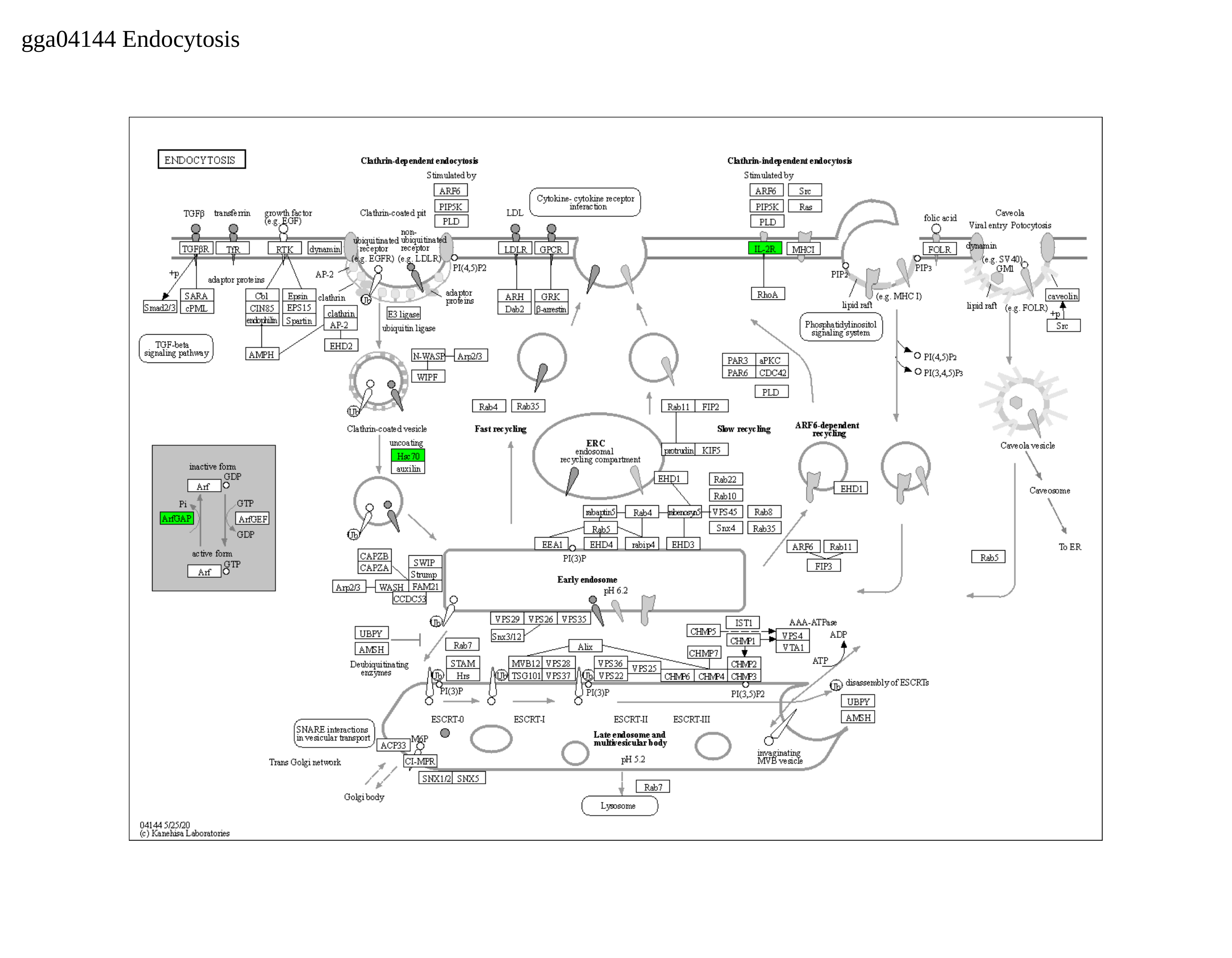

gga04144 Endocytosis

## Slide 7
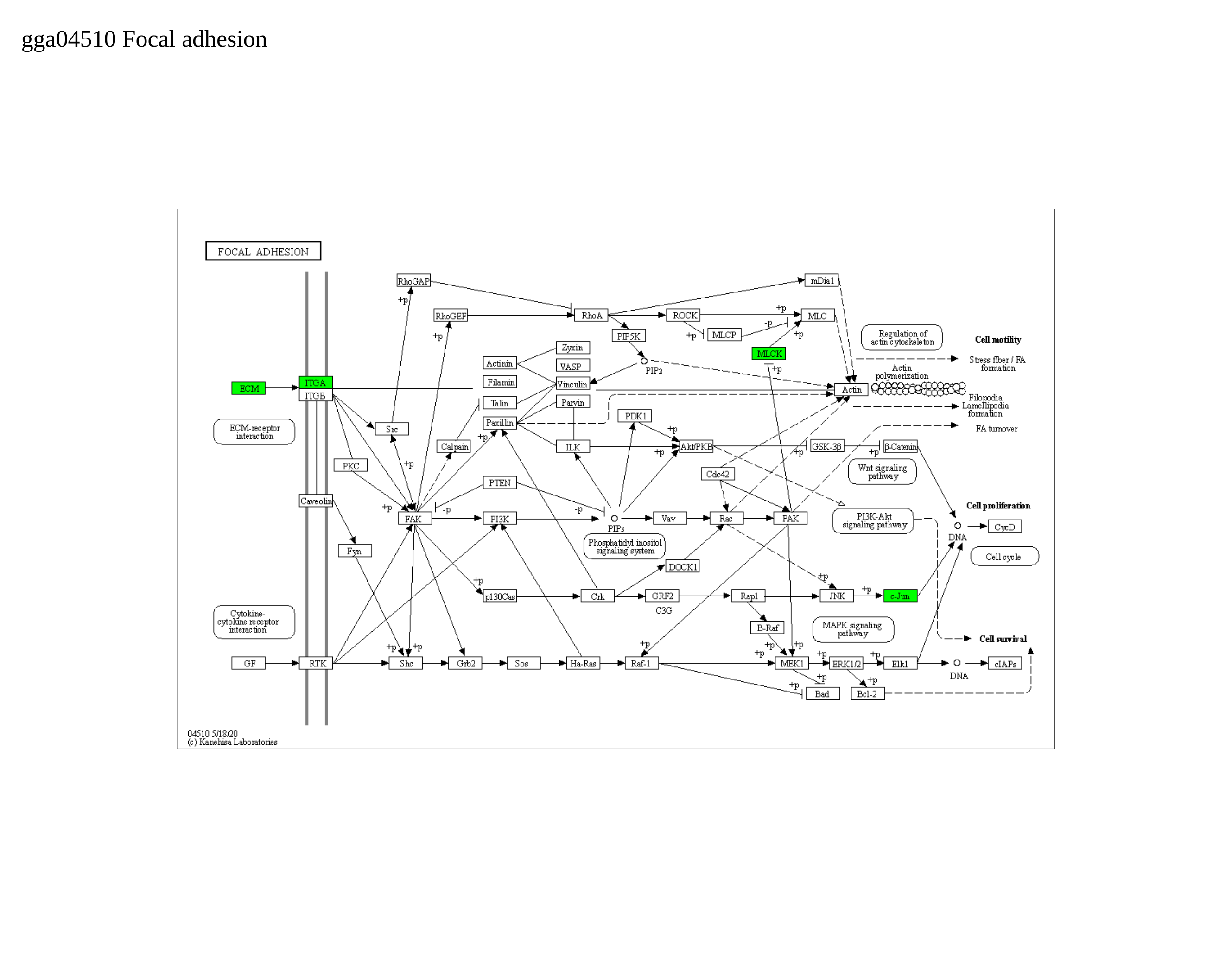

gga04510 Focal adhesion

## Slide 8
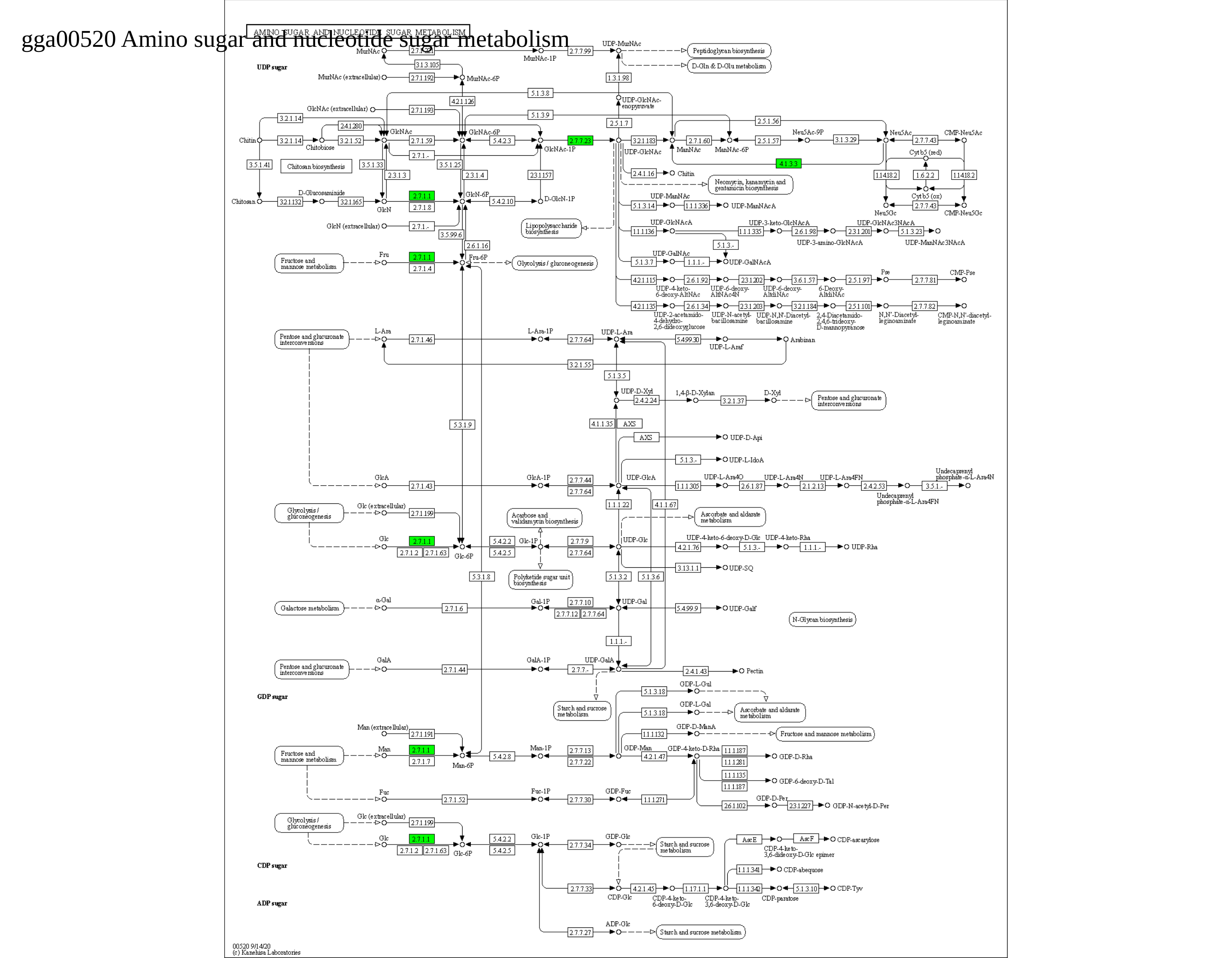

gga00520 Amino sugar and nucleotide sugar metabolism

## Slide 9
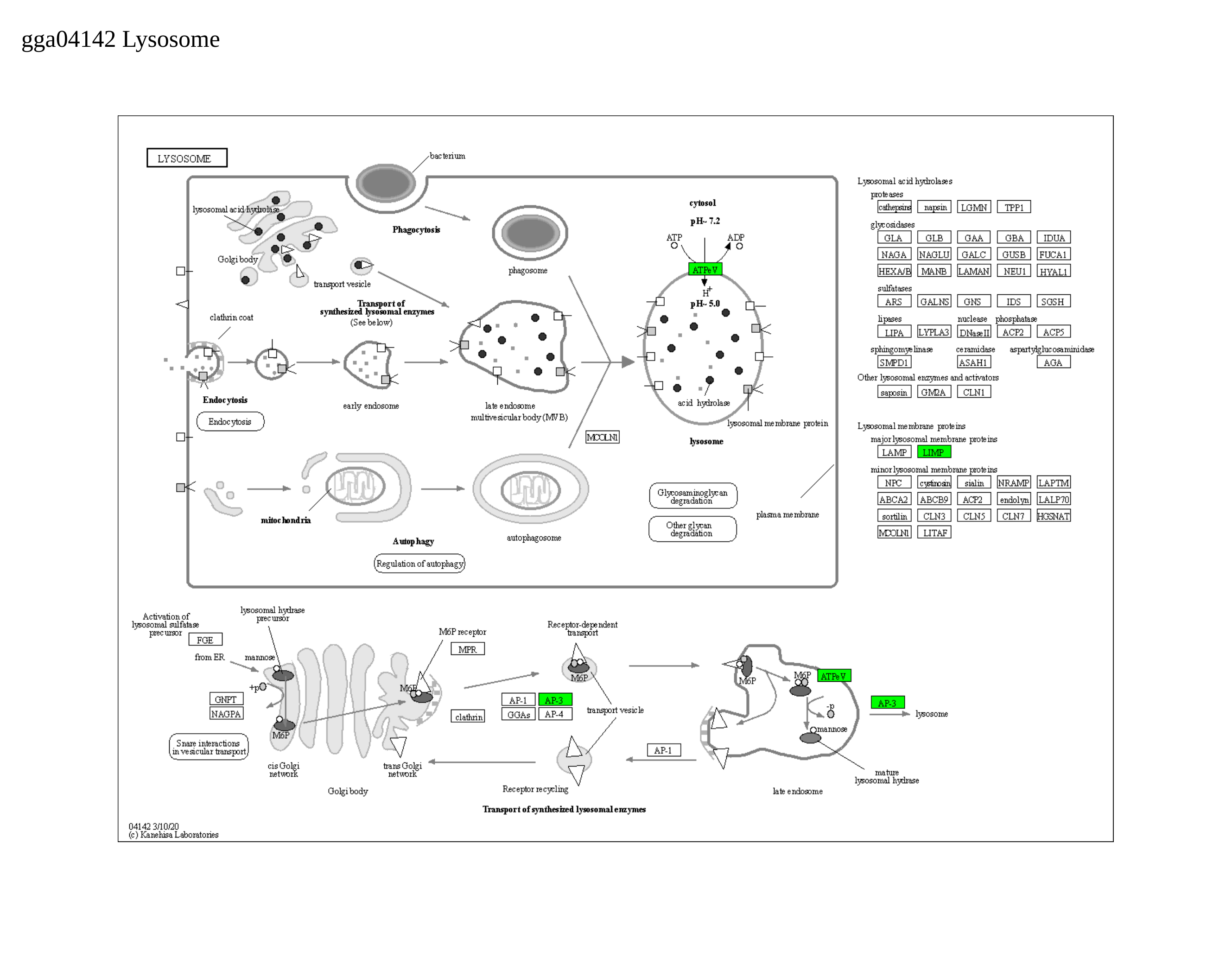

gga04142 Lysosome

## Slide 10
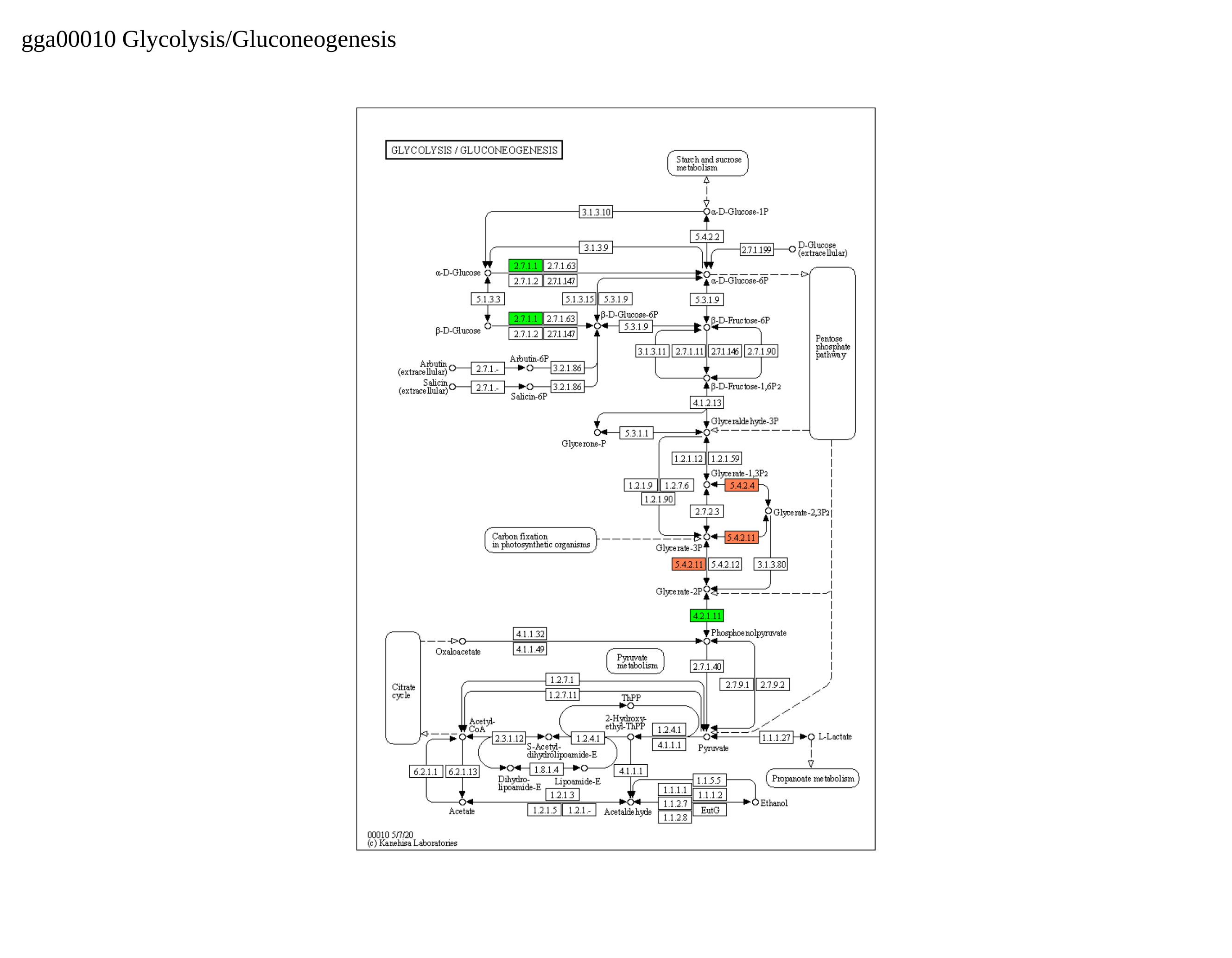

gga00010 Glycolysis/Gluconeogenesis
